# Dual PI3K/mTOR inhibitor NVP-BEZ235 induces cell cycle arrest via autophagy mediated protein degradation of RPL19 in nephroblastoma cell

**DOI:** 10.3389/fphar.2025.1588722

**Published:** 2025-07-23

**Authors:** Yan Gao, Xinran Xing, Ruizhi Cai, Dong Liu, Qili Feng, Jiaqi Luo, Yongzhao Zhu, Zeli Su

**Affiliations:** ^1^ Department of Pediatric Surgery, General Hospital of Ningxia Medical University, Yinchuan, Ningxia, China; ^2^ Clinical Medicine, Ningxia Medical University, Yinchuan, Ningxia, China; ^3^ Surgical Laboratory, General Hospital of Ningxia Medical University, Yinchuan, Ningxia, China

**Keywords:** NVP-BEZ235, PI3K, mTOR, RPL19, Autophagy

## Abstract

**Introduction:**

Nephroblastoma, the most common renal malignancy in children, is a significant health concern. NVPBEZ235, a dual inhibitor of PI3K and mTOR, has shown promise in inhibiting the growth of various cancers. However, its effects on nephroblastoma therapy are not well understood. This study aims to investigate the effects and mechanisms of NVP-BEZ235 on nephroblastoma.

**Methods:**

The proliferation of G401 cells treated with NVP-BEZ235 was evaluated using CCK-8, colony formation, and EdU assays. The effect of NVP-BEZ235 on the cell cycle was assessed by western blot and flow cytometry. To observe its impact on autophagy, protein expression and autophagic flux were examined. Bioinformatic tools were used to evaluate the expression of RPL19 in tumor tissues. The interaction between autophagy and RPL19 was also explored. In the in vivo experiments, three groups were used: NC (negative control) group, drug treatment group, and drug + RPL19 overexpression group, to assess the effect of NVPBEZ235 on tumor growth.

**Results:**

NVP-BEZ235 inhibited the proliferation of G401 cells. It arrested the cell cycle in the G2/M phase and induced autophagy. RPL19 was overexpressed in nephroblastoma tissues, and NVPBEZ235 suppressed the expression of RPL19 protein. Furthermore, the treatment with NVP-BEZ235 induced autophagy, leading to the downregulation of RPL19 expression in G401 cells. In the in vivo study, NVPBEZ235 significantly inhibited tumor growth in the drug treatment group, while RPL19 overexpression partially counteracted the drug’s effects, promoting tumor growth.

**Discussion:**

Induction of cell cycle arrest via autophagy-mediated protein degradation of RPL19 by NVP-BEZ235 effectively suppressed nephroblastoma progression. The in vivo results further suggest that the suppression of RPL19 enhances the therapeutic effects of NVP-BEZ235. These findings highlight the potential of NVP-BEZ235 as a promising therapeutic strategy for nephroblastoma, potentially through modulation of autophagy and RPL19 expression.

## 1 Introduction

Nephroblastoma is the most common pediatric kidney cancer (Wang H. et al., 2023; Zhang J. et al., 2019). The 5-year survival rate of nephroblastoma can reach to 90% under the current therapeutic patterns (Geng et al., 2022). However, approximately 20% of patients experience relapse after first-line treatment, and up to 25% of survivors report severe late treatment-related complications (Spreafico et al., 2021). Better knowing the pathogenesis and metastatic mechanisms of nephroblastoma can help to develop novel targeted treatments for such patients.

The PI3K/AKT/mTOR pathway plays a crucial role in cellular processes and is frequently dysregulated in cancers, including nephroblastoma (Akbarzadeh et al., 2021; Luo et al., 2022; Tian et al., 2022; Yu et al., 2022). Inhibiting this pathway through the use of NVP-BEZ235, which targets PI3K and mTOR, has shown promise in treating various cancers (Hongo et al., 2018; Sharma et al., 2015). And It has been demonstrated that NVP-BEZ235 effectively inhibited the growth of cisplatin-resistant urothelial cancer cell through autophagic flux (Li et al., 2013). Autophagy is also considered as a potential therapeutic target in cancer, which can be regulated by PI3K/AKT/mTOR pathway and exerts intricate effects on tumor cells (Cheng et al., 2023; Xiang et al., 2023; Yagyu and Yoshimoto, 2023). While some studies indicate that autophagy inhibits the growth of benign tumors, it has also been shown to accelerate the growth of advanced cancer (Yang et al., 2023). And when discussing the mechanism of autophagy in inhibiting tumors, it has been reported that autophagy can selectively degrade core proteins to achieve a suppressive effect on tumors (Zhang et al., 2022).

Recently, ribosomal proteins, which play essential roles in the formation of a fully functional ribosome, have been reported to be involved in cancer progression and can be employed as diagnostic and therapeutic markers for cancers (Yi et al., 2023; Yi et al., 2021). Several ribosomal proteins have been identified as surrogate markers for the activated PI3K/AKT/mTOR pathway, including ribosomal protein L19 (RPL19) (Yi et al., 2021). Due to the relationship between ribosomal protein family and PI3K/AKT/mTOR pathway, we hypothesized that NVP-BEZ235 could inhibit nephroblastoma by regulating RPL19.

In present study, we have demonstrated that NVP-BEZ235 inhibited the proliferation of G401 cells by inducing cell cycle arrest. This phenomenon is attributed to the autophagic degradation of RPL19 induced by NVP-BEZ235. Inhibition of autophagy counteracted the suppressive effect of NVP-BEZ235 on G401 cells.

## 2 Methods

### 2.1 Cell culture

G401 cells were obtained from ATCC (Shanghai, China). The cells were cultured in 10% FBS (Thermo Fisher Scientific, Waltham, United States) containing DMEM in a 37°C incubator with 5% CO_2_ and passaged every 3-4 days.

### 2.2 Tissue samples

Five paired nephroblastoma tissues and adjacent normal samples were harvested from patients (age range: 3 months to 5 years old; male: female ratio, 17:13) at the General Hospital of Ningxia Medical University. All patients had not received any anti-cancer therapy before the surgery. The diagnosed nephroblastoma tissues were reviewed by an experienced pathologist. Written informed consent was obtained from the guardians of all patients prior to sample collection. The study was approved by the Ethics Committee of the Laboratory Animal Center of Ningxia Medical University.

### 2.3 Treatment and cell transfection

NVP-BEZ235 (Cat. HY-15174, 99.87% purity) was obtained from MedChemExpress (MCE, New Jersey, United States). NVP-BEZ235 was dissolved in DMSO at a concentration of 1 mM as the primary stock and further diluted in medium. Chloroquine (CQ) and 3-methyladenine (3-MA) were purchased from Sigma-Aldrich (Saint Louis, MO, United States).

pcDNA3.1 vector was used to construct plasmid. Plasmid which expressed RPL19 was OE-RPL19 group, while pcDNA3.1 vector was used as control (OE-NC). The specific autophagy-related 5 (ATG5) short hairpin RNA (sh-ATG5) lentiviruses for ATG5 knocking down and scrambled control (sh-NC) were also constructed by the GenePharma Company (Shanghai, China). Lipofectamine 2000 (Invitrogen, Waltham, MA, United States) was used to transfection. Cells were seeded in 6-well plates at a density of 2 × 10^5 cells/well and cultured overnight to reach 70%-80% confluence. For each well, 4 μg plasmid DNA was diluted in 250 μL serum-free medium. 10 μL Lipofectamine 2000 was mixed with 250 μL serum-free medium and incubated for 5 min at room temperature. The diluted DNA and Lipofectamine 2000 were combined and incubated for 20 min at room temperature to form DNA-lipid complexes. The complexes were added dropwise to the cells. After 6 h incubation, the medium was replaced with fresh complete medium. Transfection efficiency was evaluated by western blot after 24–48 h.

### 2.4 CCK-8 assay

G401 cells were treated with different concentrations of NVP-BEZ235 (0, 25, 50, 100, 250, and 500 nM) and were added into 96-well plates (1000 per well). After incubation for different time points (0, 24, 48, and 72 h), 10 μL of CCK-8 solution (Beyotime, Shanghai, China) was added into each well. The optical density (OD) value was detected at 450 nm.

### 2.5 Clone formation assay

G401 cells were inoculated in 6-well plates for culture. After colonies were formed, methanol (Beyotime) was used to fixation, and then crystal violet (Beyotime) was used to stain.

### 2.6 5‐Ethynyl‐20‐deoxyuridine (Edu) assay

G401 cells treated with or without 100 nM NVP-BEZ235 were inoculated in 24-well plates for culture. Subsequently, EdU (Beyotime) was added for 2 h. Photographs were taken using a microscope.

### 2.7 Flow cytometric analysis

G401 cells were treated with or without NVP-BEZ235 (100 nM) for 48 h, and stained with a propidium iodide solution (Sigma). FACSCalibur flow cytometer (Becton-Dickinson, San Jose, United States) was used to cell cycle analysis.

### 2.8 qRT-PCR

PARIS™ kit (Thermo Fisher Scientific) was sued to RNAs extraction, and cDNA Synthesis Kit (TaKaRa Bio, United States) was used to reverse transcription. qRT-PCR was conducted by an ABI StepOnePlus™ System (Applied Biosystems, United States). Primers were as follows: RPL19, forward 5’- -3’: TCG​CCT​CTA​GTG​TCC​TCC​G and reverse 5’- -3’: GCG​GGC​CAA​GGT​GTT​TTT​C. 2^−△△Ct^ method was used to data treatment. The experiment conditions were as follows: 50°C for 15 min, 95°C for 15 min, 94°C for 15 s, and 57°C for 45 s, a total of 40 cycles.

### 2.9 Western blot and Co-immunoprecipitation (Co-IP)

G401 cells and tissues were lysed to extract proteins and protein separation was performed using polyacrylamide gel electrophoresis followed by protein transfer onto PVDF membranes (Millipore, United States). 5% skimmed milk was used to blocking, the membranes were incubated with antibodies against Cyclin A1 (1:1000, ab270940, Abcam, Cambridge, United States), Cyclin B1 (1:1000, ab32053, Abcam), p21 (1:1000, ab109520, Abcam), RPL19 (1:500, ab224592, Abcam), LC3, (1:1000, #12741, CST), p62 (1:1000, #39749, CST), and β-Tubullin (1:1000, #2146, CST). Then, the secondary antibody was used. ECL (Millipore) solution was used to observe the protein band. Co-IP was performed using the Pierce™ Crosslink Magnetic IP/Co-IP Kit (Thermo Scientific).

### 2.10 mRFP-GFP-LC3 adenovirus double label assay

G401 cells were transfected with mRFP‐GFP‐LC3 (Hanbio, Shanghai, China). Then, the cells were treated with NVP-BEZ235 (100 nM) or control for another 12 h. Cells were then fixed with paraformaldehyde (PFA) and nuclei were stained with DAPI (Sigma). Finally, the images of cells were taken using fluorescence microscopy.

### 2.11 Immunofluorescence assay

G401 cells treated with NVP-BEZ235 and/or 3-MA were fixed in 4% paraformaldehyde. Following by blocking with 1% BSA solution, the primary antibody of RPL19 (ab224592, Abcam) was used to incubate cells overnight at 4°C. The next day, secondary antibody was used to incubate cells for 1 h, and DAPI was used for nuclei staining for 5 min. The images were taken by Olympus FSX100 microscope (Olympus, Japan).

### 2.12 *In vivo* study

The Ethics Review Committee of Experimental Animal Welfare of the Laboratory Animal Center of Ningxia Medical University approved this study. 15 five-week-old female nude mice (BALB/C-nu/nu) were subcutaneously injected with G401 cells to establish tumor xenografts. One week after implantation, mice were treated with NVP-BEZ235 (25 mg/kg/day) or control once daily for 14 days (n = 5 each). At the end of experiment (21 days after treatment), the tumor volume was calculated as length × width^2^ × 0.5. Then, the mice were sacrificed and the tumors were taken out and weighed.

### 2.13 Bioinformatics

The mRNA expression dataset for nephroblastoma was obtained from the TARGET project in the TCGA database (https://portal.gdc.cancer.gov//). Data processing and assessment of mRNA expression were performed using R Studio.

### 2.14 Statistical analysis

The data in this study were described as mean ± standard deviation (SD). Student’s t-test or ANOVA followed by a LSD *post hoc* test was used to differences analysis using the SPSS 22.0 software. The Kaplan-Meier method was used to estimate the survival time of patients with nephroblastoma, with log-rank tests being used to assess significance. *p* < 0.05 was considered statistically significant.

## 3 Results

### 3.1 NVP-BEZ235 inhibited the proliferation of G401 cells

**FIGURE 1:**
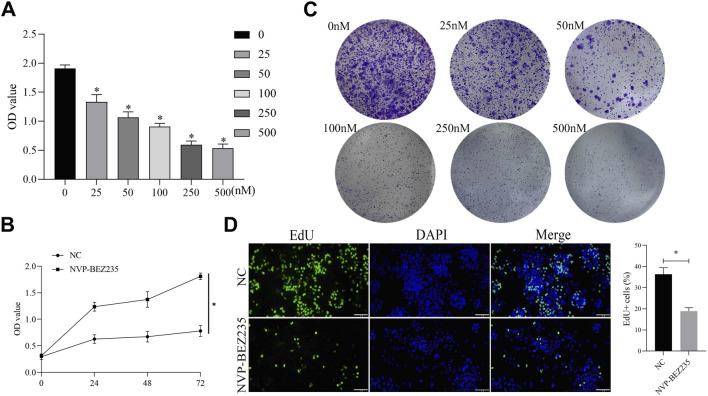
NVP-BEZ235 inhibited the growth of G401 cells. **(A)** G401 cells were treated with different concentrations of NVP-BEZ235 (0, 25, 50, 100, 250, and 500 nM) and CCK-8 assay was used to cell viability detection. **(B)** Treatment of G401 cells with NVP-BEZ235 (100 nM) for different time points followed by detection using CCK-8 assay. **(C)** The clone formation assay was conducted after G401 cells were treated with NVP-BEZ235 (0, 25, 50, 100, 250 and 500 nM). **(D)** G401 cells were treated with/without 100 nM NVP-BEZ235, and EdU assay was used to cell proliferation detection. Data are presented as mean ± SD of three independent experiments; *P < 0.05, NC: Negative Control.

In this study, we evaluated the role of NVP-BEZ235 on G401 cells. G401 cells were treated with NVP-BEZ235 (0, 25, 50, 100, 250, and 500 nM), and CCK-8 assay was used to detect the cell viability. The results clarified that NVP-BEZ235 inhibited the viability of G401 cells in a dose- and time-dependent manner (Figures 1A,B). The clone formation assay was conducted. The cloning formation rate of G401 cells was significantly reduced with the increase of NVP-BEZ235 levels (Figure 1C). And the EdU assay demonstrated that the proliferation of G401 cells was significantly attenuated after NVP-BEZ235 treatment (Figure 1D). Taken together, the above results indicated that NVP-BEZ235 could inhibit the proliferation of G401 cells.

### 3.2 NVP-BEZ235 induced cell cycle arrest

**FIGURE 2:**
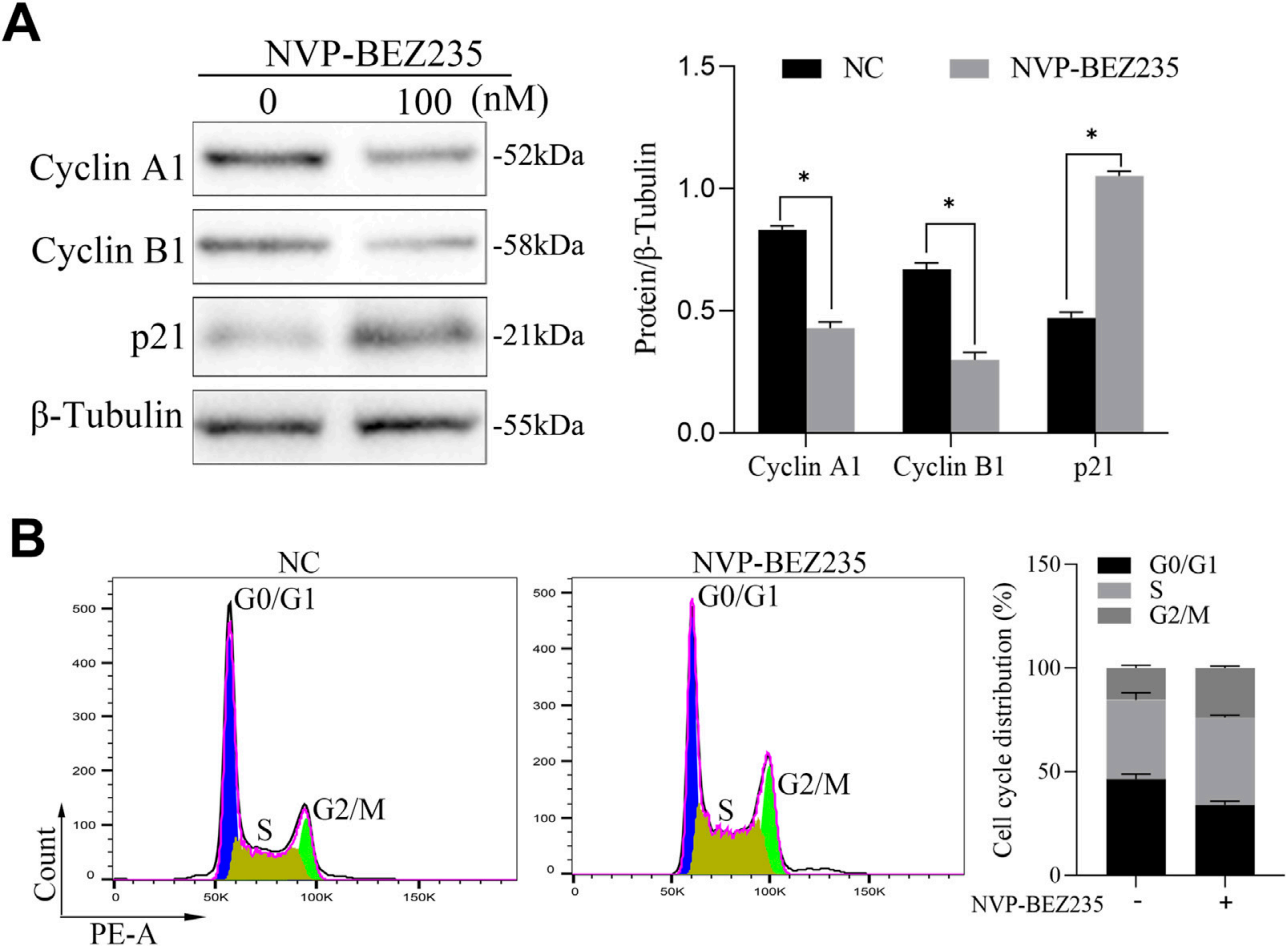
NVP-BEZ235 induced cell cycle arrest. **(A)** G401 cells were treated with NVP-BEZ235 (100 nM), and the levels of Cyclin A1, Cyclin B1, and p21 were quantified by western blot analyses. The blot shown is representative of three independent biological replicates with technical duplicates. **(B)** The cell cycle phase after NVP-BEZ235 treatment was determined by flow cytometric analysis. Data are presented as mean ± SD of three independent experiments; *P < 0.05, NC: Negative Control.

For a better understanding of the mechanism of NVP-BEZ235-induced inhibition of G401 cells, cell cycle changes were examined. G401 cells were treated with NVP-BEZ235 (100 nM), and Cyclin A1, Cyclin B1, and p21 expressions were quantified by western blot analyses. NVP-BEZ235 increased the expression of p21 and decreased the expressions of Cyclin A1 and Cyclin B1 (Figure 2A). In addition, the flow cytometric results showed that cells in the G2/M phase was increased in NVP-BEZ235 treatment (*P* < 0.05, Figure 2B). The data indicated that NVP-BEZ235 could induce cell cycle arrest at G2/M phase in G401 cells.

### 3.3 NVP-BEZ235 downregulated the expression of RPL19 at a protein level

**FIGURE 3:**
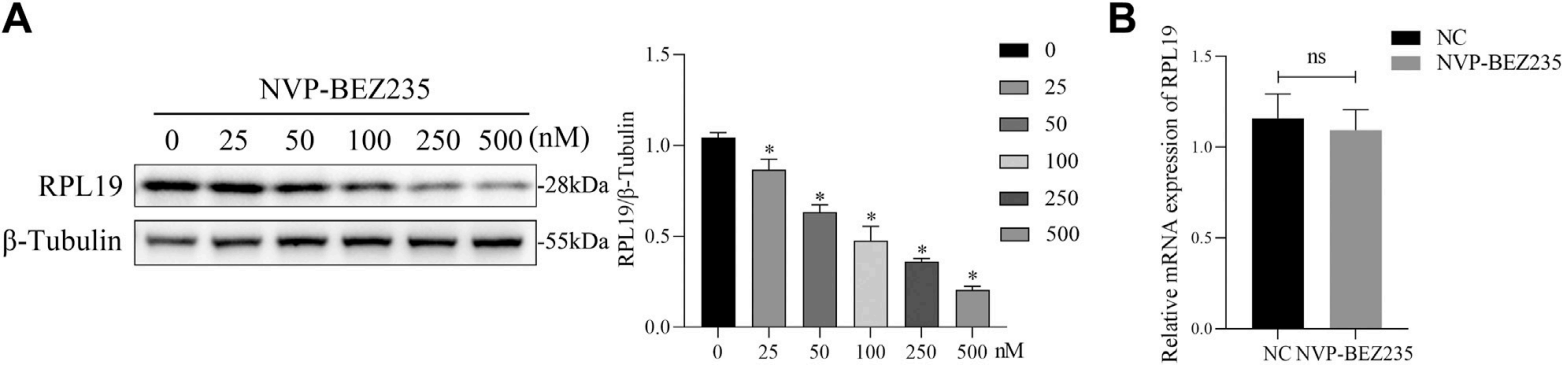
NVP-BEZ235 downregulated the expression of RPL19 at a protein level. **(A)** G401 cells were treated with NVP-BEZ235 (0, 25, 50, 100, 250, and 500 nM) and western blot was used to RPL19 expression detection. The blot shown is representative of three independent biological replicates with technical duplicates. **(B)** The mRNA levels of RPL19 after NVP-BEZ235 treatment were determined by qRT-PCR. Data are presented as mean ± SD of three independent experiments; *P < 0.05, ns: not significant, NC: Negative Control.

Previous studies have shown that NVP-BEZ235 could inhibit the activation of the downstream effectors such as ribosomal proteins (Serra et al., 2008; Xie et al., 2017). To validate whether NVP-BEZ235 could regulate the expression of RPL19, G401 cells were treated with NVP-BEZ235 (0, 25, 50, 100, 250, and 500 nM) and the expressions of RPL19 were detected. The results showed that NVP-BEZ235 dose‐dependently downregulated the expression of RPL19 at a protein level (Figure 3A). However, qRT-PCR analysis indicated that NVP-BEZ235 did not affect the expression of RPL19 at a gene level (Figure 3B). The mRNA levels of RPL19 with/without NVP-BEZ235 treatment were not significantly different (*P* > 0.05, Figure 3B). These data indicated that NVP-BEZ235 might suppress the expression of RPL19 at the protein level.

### 3.4 RPL19 was overexpressed in nephroblastoma

**FIGURE 4:**
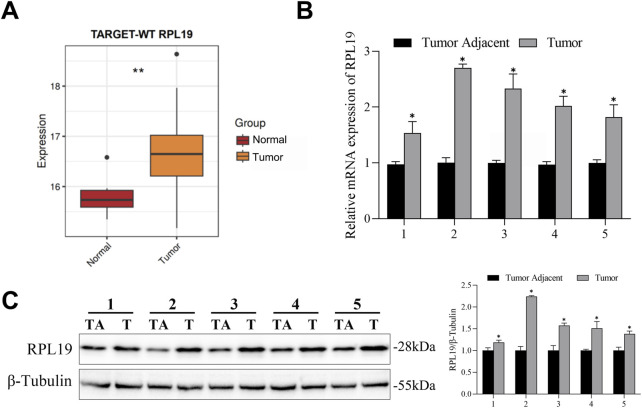
RPL19 was overexpressed in nephroblastoma. **(A)** The RPL19 mRNA expression dataset in nephroblastoma tissues and normal tissues was analyzed by R Studio. **(B)** RPL19 expression in 5 paired nephroblastoma tissues and corresponding adjacent samples was analyzed by qRT-PCR. **(C)** RPL19 expression in nephroblastoma tissues and corresponding adjacent samples was analyzed by western blot. The blot shown is representative of three independent biological replicates with technical duplicates. Data are presented as mean ± SD of three independent experiments; *P < 0.05, TA: Tumor Adjacant, T: Tumor.

The expression of RPL19 in nephroblastoma tissues and normal tissues was retrieved from the TCGA database. Bioinformatics analysis showed that RPL19 levels were much higher in nephroblastoma tissues than in normal samples (*P* < 0.05, Figures 4A). In order to confirm the expression of RPL19 in nephroblastoma, 5 paired nephroblastoma tissues and corresponding adjacent samples were collected. And the qRT-PCR analysis revealed that RPL19 expression was markedly increased in nephroblastoma tissues (*P* < 0.05, Figure 4B). Similarly, western blot results confirmed that RPL19 expression was much higher in nephroblastoma tissues than normal tissues (*P* < 0.05, Figure 4C). The above data suggested that RPL19 was overexpressed in nephroblastoma tissues.

### 3.5 RPL19 reversed the effect of NVP-BEZ235 on cell cycle

**FIGURE 5:**
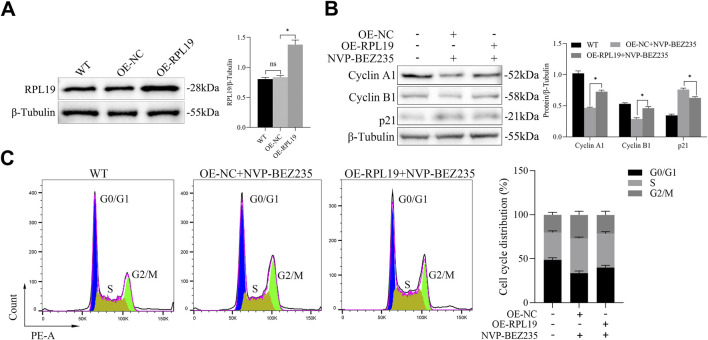
RPL19 reversed the effect of NVP-BEZ235 on cell cycle. **(A)** The transfection efficiency of OE-RPL19 in G401 cells was detected by Western blotting. **(B)** The levels of Cyclin A1, Cyclin B1, and p21 in G401 cells transfected with OE-RPL19 under the treatment of NVP-BEZ235 were detected by western blot. **(C)** The cell cycle phase in G401 cells transfected with OE-RPL19 after NVP-BEZ235 treatment was determined by flow cytometric analysis. Data are presented as mean ± SD of three independent experiments; *P < 0.05, WT: Wild Type.

To determine whether NVP-BEZ235 inhibited cell cycle in G401 cells through regulating RPL19 expression, plasmids of OE-RPL19 and control were transfected into G401. To evaluate the transfection efficiency of the RPL19 plasmid, we transfected cells and assessed RPL19 expression using Western blot. The results showed that RPL19 expression was significantly increased in the RPL19-transfected group (OE-RPL19) compared to the negative control (OE-NC) and wild-type (WT) groups, confirming successful transfection (Figure 5A). Then, we detected the levels of Cyclin A1, Cyclin B1, and p21 in G401 cells transfected with OE-RPL19 under the treatment of NVP-BEZ235. The results showed that Cyclin A1 and Cyclin B1 expressions were increased, while p21 expression was decreased in OE-RPL19 + NVP-BEZ235 group, comparing to OE-NC + NVP-BEZ235 group (*P* < 0.05, Figure 5B). The flow cytometric results showed that cells in the G2/M phase was increased in the OE-NC + NVP-BEZ235 group (*P* < 0.05, Figure 5C). However, RPL19 + NVP-BEZ235 group had fewer cells in the G2/M phase than OE-NC + NVP-BEZ235 group (*P* < 0.05, Figure 5C), indicating RPL19 exhibited a reversed role on cell cycle against NVP-BEZ235. The above data indicated that RPL19 mediated the effect of NVP-BEZ235 on cell cycle arrest in G401 cells.

### 3.6 NVP-BEZ235 induced autophagy in G401 cells

**FIGURE 6:**
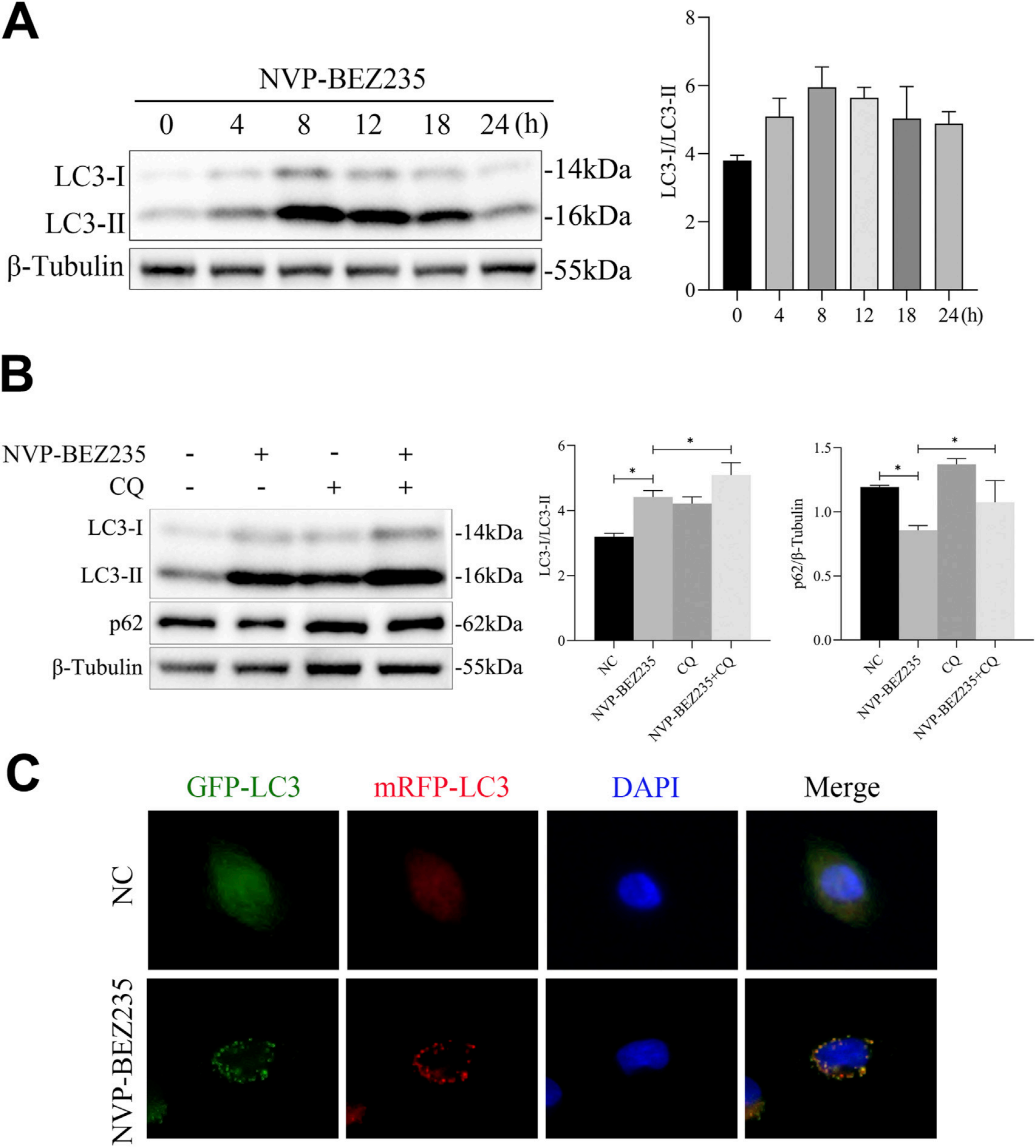
NVP-BEZ235 induced autophagy in G401 cells. **(A)** G401 cells were treated with 100 nM NVP-BEZ235 for 0, 4, 8, 12, 18, and 24 h, and western blot was used to LC3 expression detection. **(B)** G401 cells were treated with 100 nM NVP-BEZ235 and/or 5 µM CQ for 12 h, and the expressions of LC3 and p62 were quantified by western blot. **(C)** The autophagy flux was observed by transfection with mRFP-GFP-LC3 adenovirus. Data are presented as mean ± SD of three independent experiments; *P < 0.05, NC: Negative Control.

Previous evidences have shown that agents for cancer therapy that targeted mTOR can induce autophagy of cancer cells (Oh et al., 2016). Thus, in this study, we investigated whether NVP-BEZ235 could induce autophagy in G401 cells. Firstly, G401 cells were treated with 100 nM NVP-BEZ235 for 0, 4, 8, 12, 18, and 24 h, and the expression of LC3 was detected by western blot. We found that the level of LC3-II increased firstly and then decreased with the increase of the NVP-BEZ235 exposure time (Figure 6A). Then, G401 cells were treated with 100 nM NVP-BEZ235 and/or 5 µM CQ for 12 h, and the expressions of LC3 and p62 were quantified. Chloroquine (CQ) is a commonly used autophagy inhibitor that blocks autophagic flux by inhibiting lysosomal acidification, thereby preventing autophagosome-lysosome fusion and subsequent degradation, which leads to accumulation of LC3-II and p62. As shown in Figure 6B, CQ successfully blocked autophagic flux, as demonstrated by the increased expression of LC3-II and p62 in CQ treatment group compared to the control. However, NVP-BEZ235 increased the expression of LC3-II and decreased the level of p62. After treatment with CQ, the expression of p62 and LC3-II was further enhanced (Figure 6B), suggesting NVP-BEZ235 induced autophagy in G401 cells. Finally, we transfected mRFP‐GFP‐LC3 to observe the process of autophagic flux influenced by NVP-BEZ235. The results showed that NVP-BEZ235 treatment significantly increased the number of autophagosomes in G401 cells (Figure 6C). The data indicated that NVP-BEZ235 could induce autophagy in G401 cells.

### 3.7 Inhibition of autophagy attenuated the inhibition effect of NVP-BEZ235 on RPL19 expression

**FIGURE 7:**
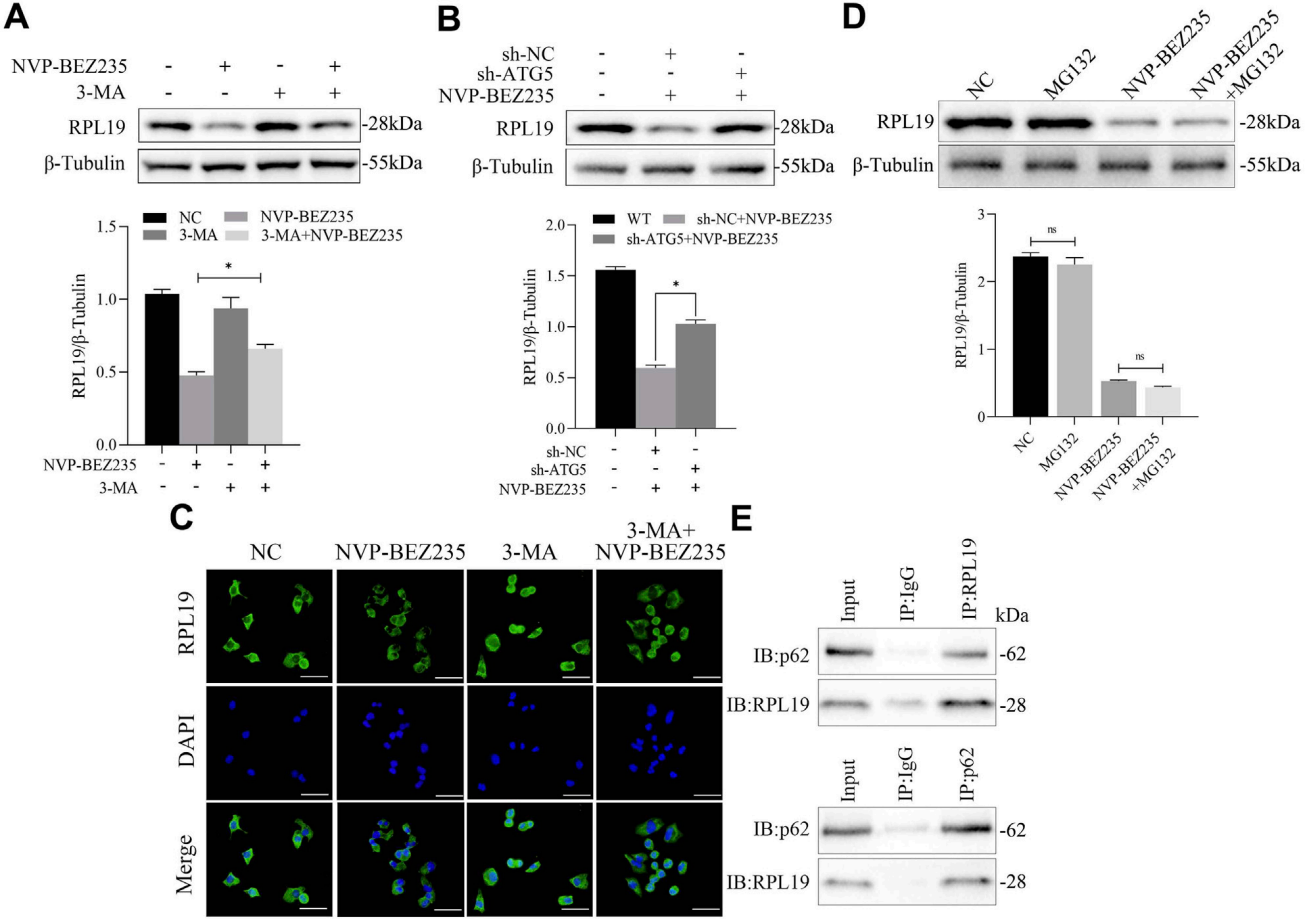
Inhibition of autophagy attenuated the inhibitory effect of NVP-BEZ235 on RPL19 expression. **(A)** G401 cells were treated with NVP-BEZ235 and/or 3-MA, and western blot was used to RPL19 expression detection. **(B)** G401 cells transfected with sh-ATG5 or sh-NC were treated with NVP-BEZ235, and the expression of RPL19 was detected by western blot. **(C)** G401 cells were treated with NVP-BEZ235 and/or 3-MA, and immunofluorescence analysis was used to RPL19 expression detection. **(D)** G401 cells were treated with NVP-BEZ235 and/or MG132, and western blot was used to RPL19 expression detection. **(E)** The interaction of RPL19 and p62 under the treatment of NVP-BEZ235 was detected by co-immunoprecipitation experiment. Data are presented as mean ± SD of three independent experiments; *P < 0.05.

To establish the functional role of the interaction between NVP-BEZ235 and RPL19 in the autophagic program of G401 cells, we used an autophagy inhibitor and sh-ATG5 to inhibit autophagy of G401 cells and observed the expression of RPL19. 3-MA is a specific autophagy inhibitor. As shown in Figure 7A, we found that using 3-MA to inhibit autophagy attenuated the inhibition effect of NVP-BEZ235 on RPL19 expression. ATG5 is a key component of autophagy, which regulates the formation of the autophagosome. In order to inhibit autophagy, G401 cells were transfected with sh-ATG5 for ATG5 knockdown. We found that the decrease of RPL19 expression in G401 cells after NVP-BEZ235 treatment were successfully abolished by ATG5 knockdown (Figure 7B). Furthermore, immunofluorescence analysis showed that 3-MA fought against NVP-BEZ235 to increase the expression of RPL19 (Figure 7C). We also investigated the potential involvement of the ubiquitin-proteasome system. Using MG132, a proteasome inhibitor, we observed that RPL19 degradation was not mediated through the ubiquitin-proteasome pathway (Figure 7D). Then, we detected the interaction of RPL19 and p62 by co-immunoprecipitation experiments, which indicated that p62 could interact with RPL19 under the treatment of NVP-BEZ235 in G401 cells (Figure 7E). The above data further confirmed that NVP-BEZ235 induced autophagy in G401 cells which resulted in p62 interaction with RPL19 leading to RPL19 protein degradation.

### 3.8 NVP-BEZ235 inhibited the growth of nephroblastoma *in vivo*

**FIGURE 8:**
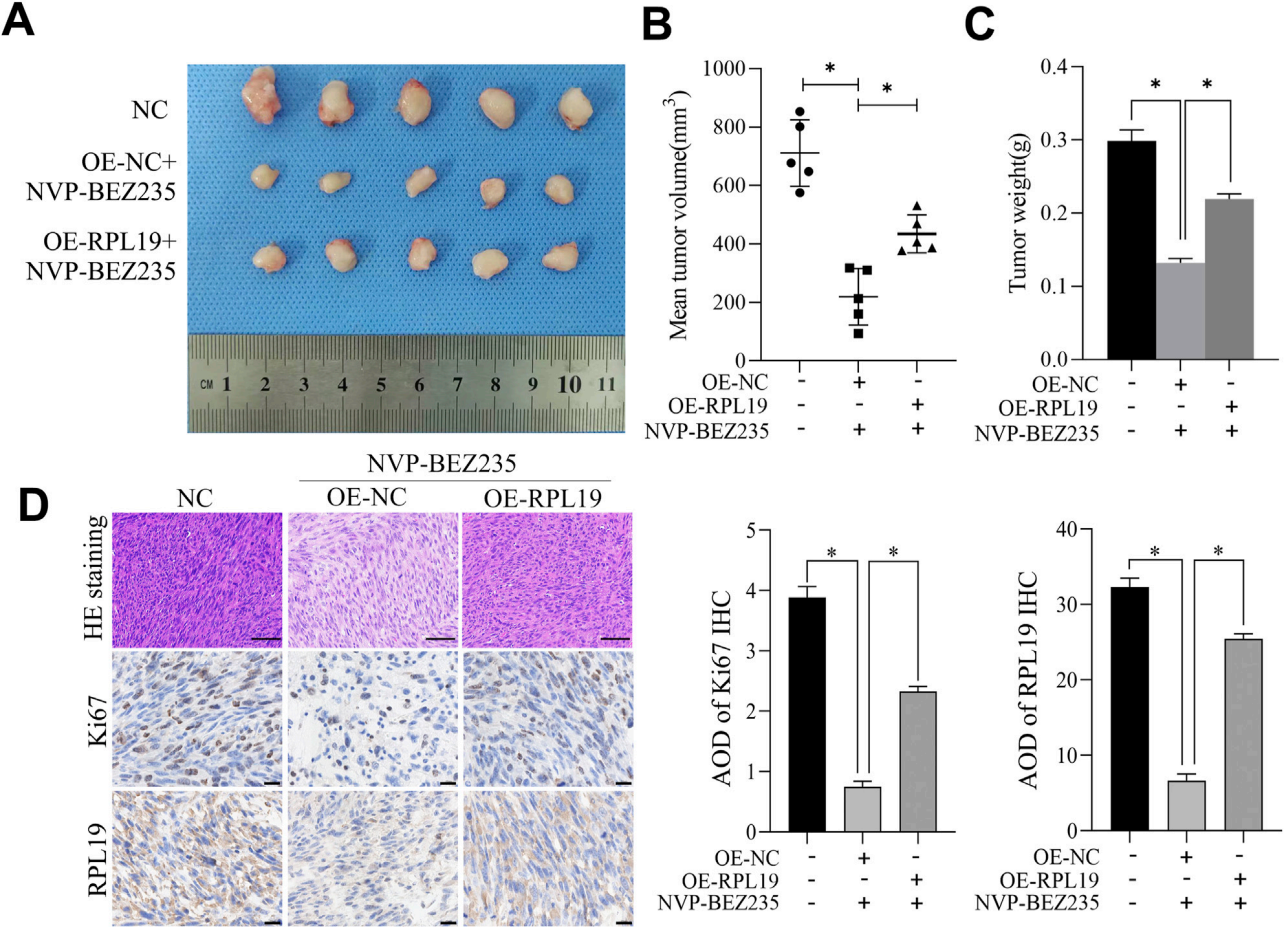
NVP-BEZ235 inhibited the growth of nephroblastoma *in vivo*. **(A)** Image of the tumor specimen. **(B)** The tumor volume in each group. **(C)** The tumor weight in each group. **(D)** Tumor tissue sections were examined using H&E staining and immunohistochemical staining for Ki67 and RPL19. Data are presented as mean ± SD; *P < 0.05, NC: Negative Control.

Tumor xenografts assay was used to investigate the effect of NVP-BEZ235 on nephroblastoma growth *in vivo*. Our results revealed that NVP-BEZ235 treatment significantly decreased tumor growth and tumor weight. However, overexpression of RPL19 reversed this effect (Figures 8A–C). Hematoxylin-eosin staining revealed that tumors treated with NVP-BEZ235 had a loose structure, with increased inflammatory cell infiltration in tumor tissues. However, this effect was reversed by overexpression of RPL19. Moreover, compared with the control group, expression levels of Ki-67 and RPL19 were lower in tumors from NVP-BEZ235 treated mice, while overexpression of RPL19 elevated the levels of both Ki-67 and RPL19 (Figure 8D).

## 4 Discussion

Nephroblastoma is an embryonal type of renal cancer, which is most observed in children (Sakthivel et al., 2023). Although the overall survival rate of children with WT is high under existing treatments, there remains a significant population at risk for disease recurrence and severe late-stage complications (Spreafico et al., 2021). Hence, it remains imperative to explore new drugs or therapeutic targets to improve the prognosis of WT patients. NVP-BEZ235 could bind to the ATP-binding clefts of PI3K and mTOR (both the mTORC1 and mTORC2) kinase, thereby inhibiting their activities (Ma et al., 2019). The PI3K/AKT/mTOR signaling mediates the processes of tumor proliferation, invasion and metastasis (Luo et al., 2022; Tian et al., 2022). NVP-BEZ235 can effectively reverse the hyper-activation of the PI3K/AKT/mTOR signaling, resulting in the antitumor activities in a broad range of cancers (Wang R. et al., 2023). Everolimus, an mTOR inhibitor similar to NVP-BEZ235, exerts its anticancer effects primarily by targeting the mTOR signaling pathway. This inhibition blocks downstream effectors like S6 ribosomal protein and 4EBP1, suppressing tumor cell proliferation, promoting apoptosis, and counteracting hyperactivation caused by oncogenic mutations. In breast cancer models, everolimus demonstrates significant antiproliferative activity with measurable IC_50_ values and exhibits synergistic effects when combined with MEK inhibitors like trametinib. However, this synergy is cell line-dependent, reflecting its reliance on crosstalk between PI3K/mTOR and ERK pathways (Leung et al., 2014). For colorectal cancer, everolimus inhibits the PI3K/AKT/mTOR axis to disrupt cellular transformation and tumor progression while reducing drug resistance, highlighting its therapeutic potential in this malignancy (Bahrami et al., 2018). In this study, We found that NVP-BEZ235 significantly inhibited the proliferation of G401 cells, exhibiting a similar inhibitory effect on G401 cells like other cancer cell lines (Ma et al., 2019; Ruan et al., 2020; Cai et al., 2020).

It has been reported that the activity of AKT and mTOR is required for the G1/S transition (Tsa et al., 2022). Thus, using NVP-BEZ235 to inhibit PI3K/AKT/mTOR signaling may result in cell cycle arrest in cancer cells. This hypothesis has been validated by several previous studies. NVP-BEZ235 was demonstrated to downregulate the expression of cell cycle positive regulators such as Cyclin A1, Cyclin B1, Cyclin D1 and upregulate the expression of p21 and p27, thus resulting in cell cycle arrest (Oh et al., 2016; Cao W. et al., 2021). Similarly, we observed that NVP-BEZ235 increased p21 expression and decreased Cyclin A1 and Cyclin B1 expression in the present study. However, different from other studies reporting the induction effect of NVP-BEZ235 on cell cycle arrest at G0/G1 phase (Cao W. et al., 2021; Li et al., 2021) or G1/S phase (Oh et al., 2016), we found that NVP-BEZ235 induced cell cycle arrest of G401 cells at G2/M phase. Different stimuli intensity or stimuli exposure time and different cell types may contribute to the above different results.

Previous studies showed that ribosomal proteins are closely associated with the activated PI3K/AKT/mTOR pathway (Yi et al., 2021). For example, Yi et al. (Yi et al., 2023) found that ribosomal protein L22‐like1 promoted prostate cancer proliferation and invasion. Cao et al. (Cao X. et al., 2021) reported that ribosomal protein L36 was a regulator of PI3K/AKT/mTOR signaling. RPL19 is a member of the ribosomal protein family. It was reported that RPL19 plays an important role in the progression of hepatocellular carcinoma (Rao et al., 2021), lung adenocarcinoma (Wei et al., 2023) and breast cancer (Hong et al., 2014). Due to the relationship between ribosomal protein family and PI3K/AKT/mTOR pathway, we investigated the effect of NVP-BEZ235 on RPL19 expression. Interestingly, we found that NVP-BEZ235 downregulated the expression of RPL19. Then, we analyzed the expression of RPL19 in nephroblastoma tissues. As expected, our data suggested that RPL19 expression was high in nephroblastoma tissues. Subsequently, we transfected OE-RPL19 in G401 cells to overexpress RPL19 and observed the changes of cell cycle phase. We found that overexpression of RPL19 reversed the effect of NVP-BEZ235 on cell cycle in G401 cells. NVP-BEZ235 might regulate RPL19 expression to induce cell cycle arrest.

Autophagy is a self-degradation system that is critical for maintaining cellular homeostasis (36). Autophagy deregulation is involved in various cancers including nephroblastoma (Li et al., 2018). Modulating autophagy activation is a potential adjuvant strategy to treat nephroblastoma (Li et al., 2018; Li et al., 2022). mTOR plays a negative role in autophagy and there are many pharmacologic molecules that can induce or inhibit autophagy via mTOR-independent mechanisms (Wang et al., 2019). As a dual inhibitor of PI3K and mTOR, NVP-BEZ235 has shown to induce autophagy in multiple myeloma cells (Ma et al., 2019), colorectal cancer cells (Oh et al., 2016), and esophageal cancer cells (Wu et al., 2018). In the present study, we also found that NVP-BEZ235 had autophagy enhancement effect in G401 cells, as demonstrated by the following evidences: (1) NVP-BEZ235 induced autophagy, increased the level of LC3-II and decreased the expression of p62 in G401 cells; (2) NVP-BEZ235 treatment significantly increased autophagosomes and autolysosomes in G401 cells. In our study,we stated that the molecular mechanism of RPL19 degradation is via autophagy. We also investigated the potential involvement of the ubiquitin-proteasome system. Using MG132, a proteasome inhibitor, we observed that RPL19 degradation was not mediated through the ubiquitin-proteasome pathway. These findings suggest that RPL19 degradation occurs primarily via autophagy.

Recently, emerging evidence highlights an interconnection between ribosomal proteins and autophagy (Pecoraro et al., 2021). Artero-Castro et al. (2015) reported that the depletion of ribosomal proteins such as uL10, RPLP1, and RPLP2 could cause an increase in autophagic occurrence. Zhang L. et al. (2019) found that RPL35 knockdown resulted in the upregulation of ATG5 expression. Here, we found that the decrease of RPL19 expression in G401 cells after NVP-BEZ235 treatment were successfully abolished by autophagy inhibition, suggesting that NVP-BEZ235 treatment could affect RPL19 expression in G401 cells through autophagy. Through co-immunoprecipitation experiments, we further found that NVP-BEZ235 regulated RPL19 expression by interacting with p62, an autophagy adaptor protein (Jiang et al., 2015). Our data preliminarily illustrated the possible mechanism how NVP-BEZ235 regulated autophagy in G401 cells.

Previously, NVP-BEZ235 has been shown to suppress the growth of xenografts generated from thyroid cancer cells (Ruan et al., 2020), hepatocellular carcinoma cells (Liu et al., 2019) and HER2-positive gastric cancer cells (Zhu et al., 2015). To investigate the effect of NVP-BEZ235 on nephroblastoma growth *in vivo*, we established tumor xenografts of nephroblastoma. Our results revealed that NVP-BEZ235 treatment significantly decreased tumor growth and tumor weight, indicating NVP-BEZ235 could inhibit nephroblastoma growth *in vivo*, consistent with our *in vitro* findings.

## 5 Conclusion

In this study, we found that NVP-BEZ235 effectively inhibited the proliferation of G401 cells, a nephroblastoma cell line. Mechanistically, NVP-BEZ235 exerted its antitumor effects by inducing cell cycle arrest at the G2/M phase and promoting autophagy in G401 cells. Our results suggest that NVP-BEZ235 could modulate RPL19 expression through autophagy, contributing to its antitumor effects. This study expands the understanding of NVP-BEZ235’s mechanism of action and highlights its potential as a novel therapeutic strategy for nephroblastoma treatment.

## Data Availability

The original contributions presented in the study are included in the article/supplementary material, further inquiries can be directed to the corresponding authors.
